# Guiding Antibiotic Therapy with Machine Learning: Real-World Applications of a CDSS in Bacteremia Management

**DOI:** 10.3390/life15111756

**Published:** 2025-11-15

**Authors:** Juan Carlos Gómez de la Torre, Ari Frenkel, Carlos Chavez-Lencinas, Alicia Rendon, Yoshie Higuchi, Jose M. Vela-Ruiz, Jacob Calpey, Ryan Beaton, Isaac Elijah, Inbal Shachar, Everett Kim, Sofia Valencia Osorio, Jason James Lee, Gabrielle Grogan, Jessica Siegel, Stephanie Allman, Miguel Hueda-Zavaleta

**Affiliations:** 1 Clinical Laboratory Roe, Lima 15076, Peru; yoshie.higuchi@labroe.com; 2 Arkstone Medical Solutions, Boca Raton, FL 33428, USA; afrenkel@arkstonemedical.com (A.F.); arendon@arkstonemedical.com (A.R.); sallman@arkstonemedical.com (S.A.); 3 Faculty of Medicine, Ricardo Palma University, Lima 15039, Peru; jose.vela@urp.edu.pe; 4 Hospital Nacional Edgardo Rebagliati Martins, Lima 15073, Peru; cchavezl1@unmsm.edu.pe; 5 Facultad de Medicina, Universidad Nacional Mayor de San Marcos, Lima 15072, Peru; 6 Department of Medicine, FAU Charles E. Schmidt College of Medicine, Boca Raton, FL 33431, USA; jcalpey2023@health.fau.edu (J.C.); rbeaton2023@health.fau.edu (R.B.); igirgis2023@health.fau.edu (I.E.); ishachar2017@fau.edu (I.S.); ekim2023@health.fau.edu (E.K.); svalenciaoso2023@health.fau.edu (S.V.O.); jasonlee2019@health.fau.edu (J.J.L.); 7 College of Science & Mathematics, University of North Georgia, Oakwood, GA 30566, USA; gegrog2775@ung.edu; 8 Lake Erie College of Osteopathic Medicine, Bradenton, FL 34211, USA; jessicasiegel6701@gmail.com; 9 Diagnóstico, Tratamiento e Investigación de Enfermedades Infecciosas y Tropicales, Universidad Privada de Tacna, Tacna 23003, Peru; mighueda@virtual.upt.pe

**Keywords:** artificial intelligence, clinical decision support systems, bacteremia, antimicrobial stewardship, machine learning

## Abstract

Bacteremia is a life-threatening condition contributing significantly to sepsis-related mortality worldwide. With delayed appropriate antibiotic therapy, mortality increases by 20% regardless of antimicrobial resistance. This study evaluated the perceived clinical utility of Artificial Intelligence (AI)-powered Clinical Decision Support Systems (CDSSs) (OneChoice and OneChoice Fusion) among specialist physicians managing bacteremia cases. A cross-sectional survey was conducted with 65 unique specialist physicians from multiple medical specialties who were presented with clinical vignettes describing patients with bacteremia and 90 corresponding AI-CDSS recommendations. Participants assessed the perceived helpfulness of AI decision-making, the impact of AI recommendations on their own clinical judgment, and the concordance between AI recommendations and their own clinical judgment, as well as the validity of changing therapy based on CDSS recommendations. The study encompassed a diverse range of bacterial pathogens, with *Escherichia coli* representing 38.7% of the isolates and 30% being extended-spectrum β-lactamase (ESBL) producers. Findings show that 97.8% [(95% CI: 92.2–99.7%)] of physicians reported that AI facilitated decision-making and substantial concordance (87.8% [95% CI: 79.2–93.7%; Cohen’s κ = 0.76]) between AI recommendations and physicians’ therapeutic recommendations. Stratification by pathogen revealed the highest concordance for *Escherichia coli* bacteremia (96.6%, 28/29 cases). Implementation analysis revealed a meaningful clinical impact, with 68.9% [(95% CI: 58.3–78.2%)] of cases resulting in AI-guided treatment modifications. These findings indicate that AI-powered CDSSs effectively bridge critical gaps in infectious disease expertise and antimicrobial stewardship, providing clinicians with evidence-based therapeutic recommendations that can be integrated into routine practice to optimize antibiotic selection, particularly in settings with limited access to infectious disease specialists. For optimal clinical integration, we recommend that clinicians utilize AI-CDSS recommendations as an adjunct to clinical judgment rather than a replacement, particularly in complex cases involving immunocompromised hosts or polymicrobial infections. Future research should prioritize prospective clinical trials that evaluate direct patient outcomes to establish evidence of broader clinical effectiveness and applicability across diverse healthcare settings.

## 1. Introduction

Bacteremia represents a life-threatening medical illness with substantial global health implications, contributing significantly to sepsis-related mortality worldwide. Recent epidemiological analyses from the Global Burden of Disease Study revealed that sepsis accounts for an estimated 48.9 million incident cases annually and results in 11.0 million deaths globally, representing 19.7% of all global deaths [1]. Clinical outcomes vary substantially by pathogen, with marked differences in mortality rates across bacterial species. While Escherichia coli bacteremia demonstrates relatively lower 30-day mortality (12.1%), other pathogens show significantly higher rates, including Staphylococcus aureus (22.8%), Pseudomonas species (24.7%), and Enterococcus species (23.6%). The most striking mortality rates are observed with Clostridium species (41.9%), Candida species (32.0%), and Bacteroides fragilis (25.3%), representing more than a three-fold difference compared to *E. coli* [2,3,4]. These substantial variations in mortality underscore the critical importance of pathogen-specific risk stratification and targeted therapeutic approaches in bloodstream infections. Similarly, *Escherichia coli* bloodstream infections, despite being among the most common pathogens with incidence rates of 50–60 cases per 100,000 population, carry 30-day case-fatality rates of approximately 10–15% [2]. These epidemiological data points underscore the critical need for optimized therapeutic interventions in managing bacteremia.

Providing rapid and appropriate antibiotic treatment is crucial for effectively managing bacteremia and has a significant impact on patient outcomes, regardless of the presence of antimicrobial resistance. Extensive studies of healthcare databases have demonstrated the critical effect of antibiotic timing. Delays in administering appropriate antibiotics increase in-hospital mortality by 20%. These delays also prolong hospital stays by 70% and raise total inpatient costs by 65%. Importantly, these effects occur regardless of antimicrobial resistance status [3]. Patients with multidrug-resistant bloodstream infections face additional challenges; for example, extended-spectrum β-lactamase-producing Enterobacterales have a higher mortality rate (adjusted hazard ratio, 1.63; 95% CI, 1.13–2.35). Deaths attributable to third-generation cephalosporin-resistant *Escherichia coli* infections in the European Union and European Economic Area (EU/EEA) increased dramatically from 2139 in 2007 to approximately 8750 in 2015—equivalent to 1.7 deaths per 100,000 population—representing more than a four-fold rise over this eight-year period [5]. Evidence from meta-analyses emphasizes the importance of prompt, appropriate treatment, with molecular rapid diagnostic testing significantly reducing mortality risk (OR 0.66, 95% CI 0.54–0.80) and decreasing the time to effective therapy by approximately 5 h (95% CI −8.60 to −1.45 h) [6].

Integrating artificial intelligence (AI) and machine learning (ML) into clinical decision support systems (CDSSs) marks a transformative shift in the management of infectious diseases, significantly improving diagnostic accuracy and treatment precision. These systems have shown notable clinical benefits, including sepsis prediction algorithms that reduce mortality by 30–60% in quasi-experimental studies and 58% in randomized controlled trials. They also enable blood culture collection and antibiotic administration roughly 2.8 h earlier than traditional methods [7]. Thanks to AI’s ability to analyze complex clinical data and provide real-time, patient-specific guidance, ML-based CDSS tools are valuable for antimicrobial stewardship, especially in resource-limited settings where infectious disease specialists may be scarce [7,8].

Arkstone’s machine learning-based clinical decision support system has shown excellent performance across various internal validation tests. Using methods such as k-fold cross-validation, random subsampling, and holdout validation, the system achieved 100% accuracy in differentiating between trained and untrained single data points in 1110 tests involving 111 bacterial species and resistance genes [9]. Further evaluation with 1401 real lab results from 66 labs in 55 regions confirmed perfect precision and recall (1.0 for both), with no false positives or negatives [9]. Human-in-the-loop validation also found a 0% significant discrepancy rate compared to clinical guidelines, with only 15.53% minor discrepancies mainly related to antibiotic choices or dosing, confirming the system’s consistency with established infectious disease standards [9].

Further analysis comparing AI-driven therapeutic suggestions for bacteremia treatment has confirmed the usefulness of molecular-based decision-support systems. The assessment of Arkstone’s OneChoice platform revealed strong agreement between recommendations based solely on molecular data and those incorporating phenotypic susceptibility results (Cohen’s Kappa, 0.80), which included guidance on antibiotic selection, dosage, and treatment duration, with recommendations provided approximately 29 h earlier (median, 16.81 versus 46.32 h) [10]. For *Escherichia coli* bacteremia, the most common pathogen accounting for 41% of cases, the agreement on recommendations was 95%, highlighting the accuracy of AI-assisted antimicrobial decisions [10].

Despite advances and proven analytical accuracy, the practical utility of these decision support tools in real-world clinical settings remains unclear. This study aimed to explore how specialist physicians perceive the clinical utility of an AI-powered CDSS (OneChoice and OneChoice Fusion). The study focuses on physicians’ interpretation of the CDSS recommendation, its impact on the selection of therapy, and whether the CDSS recommendation aligns with their own clinical judgment in routine practice.

## 2. Materials and Methods

### 2.1. Study Design and Setting

This study employed a cross-sectional survey design to evaluate the perceived clinical utility of AI-powered CDSSs among specialist physicians managing cases of bacteremia. The investigation was conducted at Roe Laboratory, Lima, Peru, and involved healthcare professionals from multiple medical specialties, some with expertise in infectious disease management and others without. The study protocol was designed to assess real-world clinical recommendations by CDSSs and determine if physicians presented with the same clinical vignette would recommend the same treatment.

### 2.2. Study Population and Participants

A total of 65 unique specialist physicians were enrolled in this study, using purposive sampling, and participated in 90 survey evaluations, with some physicians evaluating multiple clinical vignettes. Physicians were selected based on their active clinical practice in managing patients with positive blood culture results and their specialization in relevant medical disciplines. All participants possessed clinical experience in interpreting blood culture results and making antimicrobial therapy treatment decisions.

#### 2.2.1. Inclusion Criteria

(1) Board-certified specialists in relevant medical disciplines; (2) active clinical practice involving bacteremia management; (3) willingness to participate in the survey evaluation; and (4) familiarity with blood culture interpretation and antimicrobial prescribing practices.

#### 2.2.2. Exclusion Criteria

(1) Physicians without active clinical practice; (2) incomplete survey responses; and (3) specialists without experience in antimicrobial therapy decision-making.

### 2.3. Clinical Decision Support Systems Evaluated

Two AI-powered CDSSs developed by Arkstone Medical Solutions were evaluated in this study.

#### 2.3.1. OneChoice System

The OneChoice system is an AI-driven platform that generates therapeutic recommendations based exclusively on molecular diagnostic data, along with patient-specific information obtained from blood culture identification panels. The system utilizes ML algorithms trained on extensive real-life clinical databases to provide real-time, pathogen-specific antimicrobial guidance that could incorporate patient-specific clinical information, including hepatic failure status, renal function, and other relevant clinical parameters (Supplement S1).

#### 2.3.2. OneChoice Fusion System

The OneChoice Fusion system is an enhanced version of the AI-CDSS that integrates both molecular diagnostic results and conventional phenotypic susceptibility testing data, along with patient-specific information to generate refined therapeutic recommendations. This system combines rapid molecular identification with traditional antimicrobial susceptibility testing profiles to optimize treatment suggestions (Supplement S2).

### 2.4. Data Collection and Survey Methodology

Data collection was conducted using a structured survey instrument administered to participating physicians in accordance with a standardized protocol. The clinical scenarios presented in the survey were developed from anonymized real-world bacteremia cases obtained from Roe Laboratory’s microbiology database. Each case reflected authentic pathogen identifications and susceptibility profiles representative of routine clinical practice. A multidisciplinary panel composed of infectious disease physicians and clinical microbiologists selected and refined these vignettes to ensure clinical relevance, diversity of organisms, and accuracy of diagnostic data. All cases were de-identified before inclusion to protect patient confidentiality. Each participant was presented with authentic positive blood culture cases and corresponding AI-CDSS recommendations in a sequential manner.

#### 2.4.1. Phase 1

Participants received initial molecular blood culture identification results accompanied by OneChoice system recommendations for antimicrobial therapy.

#### 2.4.2. Phase 2

Subsequently, conventional susceptibility testing results were provided alongside OneChoice Fusion system recommendations.

The survey instrument evaluated multiple dimensions of clinical utility, including: (1) perceived helpfulness of AI-generated information; (2) impact on therapeutic decision-making processes; (3) concordance between AI recommendations and physician clinical judgment; and (4) implementation of therapy changes based on AI guidance.

### 2.5. Microbiological Characteristics and Pathogen Distribution

The study encompassed a diverse spectrum of bacterial pathogens commonly encountered in cases of clinical bacteremia. The pathogen distribution included *Escherichia coli* (38.7% of isolates), representing the most frequently identified organism, followed by *Pseudomonas aeruginosa* (13.3%) and *Salmonella typhi* (4%). Notably, 30% of bacterial isolates were confirmed as producers of CTX-M extended-spectrum beta-lactamase, reflecting the contemporary antimicrobial resistance patterns encountered in clinical practice.

#### 2.5.1. Molecular Testing

Positive blood culture samples underwent rapid molecular analysis using the FilmArray Blood Culture Identification (BCID) Panel (BioFire Diagnostics, LLC, Salt Lake City, UT, USA) or Xpert^®^ MRSA/SA Blood Culture (Cepheid LLC, Sunnyvale, CA, USA), based on Gram stain results. The assays were conducted according to the manufacturer’s instructions, with specific attention to reagent preparation, sample volume (200 µL), and assay run conditions (temperature and duration). Extended-spectrum β-lactamase genotypes, specifically CTX-M, were identified using the FilmArray BCID Panel, which employs multiplex PCR technology to directly detect resistance genes in positive blood culture broths. The assay specifically detects CTX-M group resistance determinants with high sensitivity and specificity as validated by the manufacturer.

#### 2.5.2. Phenotypic Testing

Organisms isolated from positive blood cultures were identified on various agar media, including Blood Agar, Chocolate Agar, MacConkey Agar, and Sabouraud Agar. Microbial identification was performed using the MALDI-TOF mass spectrometry system, which was calibrated daily to ensure accuracy. Antimicrobial susceptibility testing (AST) was performed using the VITEK 2.0 automated system, in accordance with the Clinical and Laboratory Standards Institute (CLSI) [11] guidelines and interpretive criteria. AmpC β-lactamase Detection: AmpC β-lactamase production was identified through phenotypic testing using cefoxitin screening. Inducible AmpC (iAmpC) was noted when relevant organisms demonstrated phenotypic patterns consistent with inducible resistance mechanisms.

Multidrug resistance (MDR) was defined, according to the criteria established by Magiorakos et al. (2012), as acquired non-susceptibility to at least one agent in three or more antimicrobial categories relevant to the tested bacterial species [12].

### 2.6. Data Analysis

Survey responses were analyzed using descriptive statistics to characterize participant demographics, clinical specialties, and response patterns. Categorical variables were expressed as frequencies and percentages. The utility and impact assessments were quantified through response rate calculations and preference evaluations.

Statistical analyses were performed to determine the proportion of respondents who found AI systems helpful, the percentage reporting that AI facilitated decision-making, the concordance rate between AI recommendations and physician choices, and the frequency of therapy modifications guided by AI. All proportions are reported with 95% Clopper-Pearson (exact binomial) confidence intervals to provide precise estimates of uncertainty.

Adjustment for Clustering: Given that 65 physicians completed 90 surveys, with some physicians potentially contributing multiple responses, we accounted for potential clustering effects in our analysis. We estimated the intraclass correlation coefficient (ICC) at approximately 0.10–0.15, typical for healthcare survey data where responses may be correlated within providers. To address this non-independence, we calculated both unadjusted and cluster-adjusted confidence intervals. The cluster-adjusted confidence intervals are slightly wider, reflecting the reduced effective sample size due to within-physician correlation. All primary confidence intervals reported account for this clustering structure.

Cohen’s kappa (κ) with 95% confidence intervals was calculated to assess agreement between AI recommendations and physician therapeutic choices beyond chance, with values >0.60 indicating substantial agreement and >0.80 indicating almost perfect agreement according to Landis and Koch criteria.

Stratified analyses were performed by medical specialty and pathogen type to explore variations in concordance rates across clinician groups and clinical scenarios.

### 2.7. Ethical Considerations

The Faculty of Health Sciences Ethics Committee at the Universidad Privada de Tacna approved the study protocol. This study was conducted in accordance with the principles outlined in the Declaration of Helsinki for ethical medical research. All survey responses were anonymized to protect participant identity, and participation was entirely voluntary with implied consent obtained through survey completion.

### 2.8. Data and Materials Availability

All survey instruments, data collection protocols, and analytical methodologies employed in this study are available upon reasonable request to facilitate replication and further research. The AI-CDSSs evaluated (OneChoice and OneChoice Fusion) are proprietary systems developed by Arkstone Medical Solutions, with technical specifications and algorithmic details subject to intellectual property considerations. Aggregated survey data supporting the study conclusions will be made available through appropriate data-sharing mechanisms while maintaining participant confidentiality.

## 3. Results

### 3.1. Participant Demographics and Clinical Characteristics

#### 3.1.1. Specialist Physician Distribution

**Figure 1:**
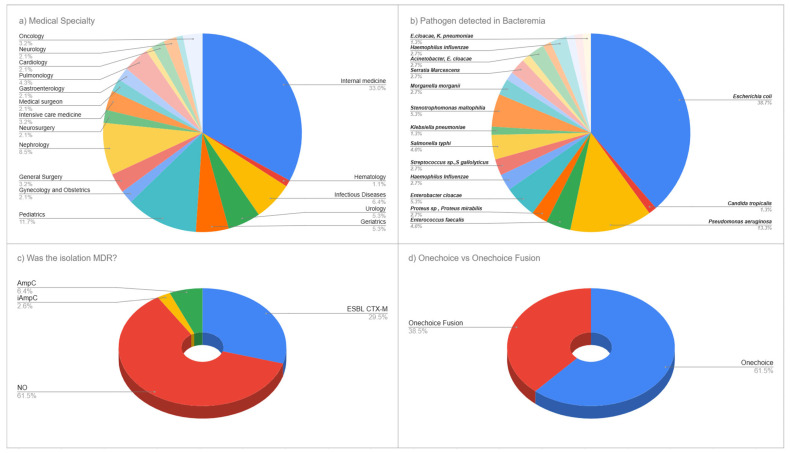
Medical Specialties of the Participants and Pathogens Detected in Bacteremia Episodes. (**a**) Distribution of medical specialties among 65 participating physicians. (**b**) Pathogen distribution among 90 bacteremia cases. (**c**) Antimicrobial resistance patterns including ESBL CTX-M (30.0%), AmpC (6.3%), and non-MDR isolates (61.3%). (**d**) Percentage of surveys associated with OneChoice vs OneChoice Fusion by participants.

A total of 65 unique specialist physicians participated in this cross-sectional survey, completing 90 total survey evaluations of clinical vignettes. Medicine specialists comprised the largest group, at 33.0% (n = 31), followed by pediatric specialists at 11.7% (n = 11), nephrology specialists at 8.5% (n = 8), and infectious disease specialists at 6.4% (n = 6). Urology and geriatrics specialists each represented 5.3% (n = 5). Pulmonology specialists made up 4.3% (n = 4). Both gastroenterology and intensive care medicine specialists constituted 3.2% (n = 3) each. Additionally, 19.1% (n = 14) of participants came from each of several other specialties, including hematology, neurosurgery, surgical medicine, general surgery, gynecology-obstetrics, cardiology, neurology, oncology, and family and community medicine. This varied specialty participation provided a broad range of clinical expertise relevant to the management of bacteremia (Figure 1a).

#### 3.1.2. Bacteremia Pathogen Characteristics

The microbiological analysis of positive blood cultures revealed a diverse range of pathogens typical of modern clinical bacteremia. *Escherichia coli* was the most common, making up 38.7% (n = 29) of all isolates, followed by *Pseudomonas aeruginosa* at 13.3% (n = 10). *Enterobacter cloacae* accounted for 5.3% (n = 4), while *Enterococcus faecalis* and *Salmonella typhi* each constituted 4.0% (n = 3). The *Salmonella typhi* cases were identified during the study period (2023–2024) and reflect the endemic burden of typhoid fever in Peru, where the disease remains a significant public health concern, particularly in areas with limited access to safe water and sanitation. Multiple organisms were found, each at 2.7% (n = 2), including *Proteus* species/*Proteus mirabilis*, *Haemophilus influenzae*, *Streptococcus* species/*Streptococcus gallolyticus*, and *Morganella morganii*. Less common pathogens included *Klebsiella pneumoniae* and *Candida tropicalis*, each accounting for 1.3% (n = 1). Additional minor pathogens included *Serratia marcescens*, *Bacteroides fragilis*, *Staphylococcus* species, the *Acinetobacter*/*Enterobacter cloacae* complex, *Citrobacter freundii*, and organisms within the Enterobacteriaceae complex, reflecting the complexity of microbiological profiles observed in current bacteremia cases (Figure 1b).

#### 3.1.3. Antimicrobial Resistance Patterns

Analysis of antimicrobial resistance showed a notable prevalence of resistance within the study group. Most isolates (61.3%, n = 55) did not display multidrug resistance; however, the production of CTX-M extended-spectrum beta-lactamase (ESBL) was seen at 30.0% (n = 27). AmpC beta-lactamase was found in 6.3% (n = 6) of isolates, and inducible AmpC resistance mechanisms were present in 2.5% (n = 2). This corresponds to an ESBL prevalence of 29.5% among all typed isolates, reflecting the contemporary challenge of antimicrobial resistance in our clinical setting. This pattern highlights the clinical complexity of managing bacteremia today and suggests that AI-guided treatment recommendations could improve antimicrobial choice for both susceptible and resistant pathogens (Figure 1c).

### 3.2. Clinical Utility Assessment of the OneChoice System

#### 3.2.1. Perceived Helpfulness and Decision-Making Impact

**Figure 2:**
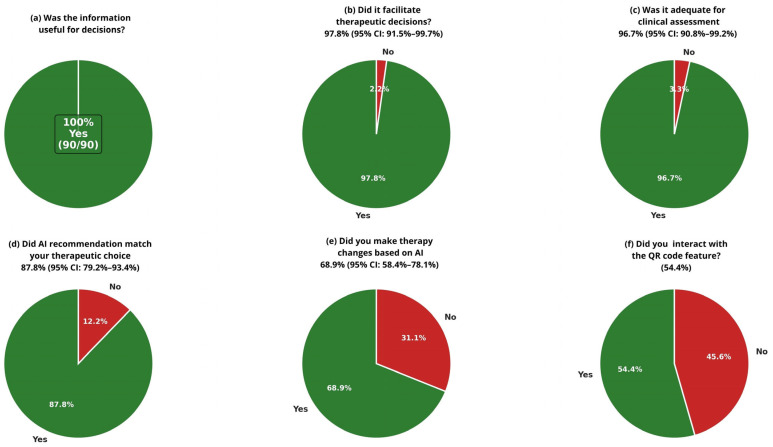
Clinical Utility Assessment of Al-CDSS (OneChoice and OneChoice Fusion Physician Survey Responses (n = 90 surveys from 65 unique physicians). Questions asked to specialist doctors who received positive molecular or conventional blood culture results, accompanied by OneChoice and OneChoice Fusion (CDSSs). (**a**–**c**) Perceived utility assessments showing that 100% found information helpful, 97.8% said that it facilitated decision-making, and 96.7% found it adequate for assessment. (**d**,**e**) Concordance (87.8%) and implementation (68.9%) rates. (**f**) QR code interaction adoption (54.4%).

The evaluation of the OneChoice system’s utility showed strong acceptance among physicians. All respondents (100%, n = 90; 95% CI: 96.0–100%) found the information helpful for clinical decisions. Its influence on therapeutic choices was also notable, with 97.8% (n = 88; 95% CI: 91.5–99.7%; cluster-adjusted 95% CI: 87.0–99.9%) of participants stating that it aided their decision-making. Most physicians, 96.7% (n = 87 [95% CI: 90.8–99.2%]), rated the AI-generated guidance as adequate for a thorough clinical assessment. These results highlight high user satisfaction and perceived clinical value across the entire group of doctors (Figure 2a–c).

#### 3.2.2. Clinical Concordance and Implementation Patterns

Assessment of clinical concordance between AI recommendations and physician therapeutic preferences showed strong alignment in decision-making. The concordance rate between OneChoice recommendations and physicians’ choices was 87.8% (n = 79 [95% CI: 79.2–93.4%; cluster-adjusted 95% CI: 75.0–94.5%]), indicating a high level of agreement between AI guidance and clinical judgment. Cohen’s kappa coefficient (κ = 0.76, 95% CI: 0.63–0.89) demonstrated substantial agreement beyond chance, confirming that the concordance reflects accurate alignment rather than random agreement. Analysis of implementation revealed a meaningful clinical effect, with 68.9% (n = 62 [95% CI: 58.4–78.1%; cluster-adjusted 95% CI: 55.0–80.0%]) of cases resulting in treatment changes, according to OneChoice data. Additionally, physician confidence in AI recommendations was evident, with 85.6% (n = 77 [95% CI: 76.6–92.1%]) of participants willing to follow OneChoice’s advice in routine practice. This demonstrates high trust in the system’s suggestions and a readiness to incorporate AI guidance into standard patient care (Figure 2d,e).

#### 3.2.3. Stratified Analysis by Specialty and Pathogen

Stratification by medical specialty revealed essential variations in concordance rates. Non-infectious disease specialists demonstrated consistently high concordance, with internal medicine (89.7%, 26/29), pediatrics (90.0%, 9/10), nephrology (100%, 8/8), geriatrics (100%, 5/5), urology (100%, 5/5), and pulmonology (100%, 4/4) all showing substantial agreement with AI recommendations. In contrast, infectious disease specialists demonstrated lower concordance (33.3%, 2/6), reflecting their advanced expertise and consideration of complex clinical factors beyond the CDSS input parameters.

Analysis of discordant cases (n = 11, 12.2%) revealed several distinct patterns. Infectious disease specialists accounted for 4 of 11 discordant cases, often preferring broader-spectrum coverage or alternative agents based on institutional antibiograms or patient-specific risk factors not captured in the CDSS input.

Pathogen-stratified analysis revealed that concordance was highest for *Escherichia coli* bacteremia (96.6%, 28/29), the most prevalent pathogen in our cohort, supporting the AI system’s particular accuracy for this common clinical scenario. *Salmonella typhi* (100%, 3/3), *Enterobacter cloacae* (100%, 3/3), and *Stenotrophomonas maltophilia* (100%, 3/3) also showed complete concordance, though sample sizes were smaller. Lower concordance rates were observed for *Enterococcus faecalis* (33.3%, 1/3) and *Serratia marcescens* (0%, 0/2)

### 3.3. Comparative Analysis: OneChoice Versus OneChoice Fusion

#### Digital Engagement and Interactive Features

Physicians adopted the interactive features of AI-CDSS platforms to a moderate degree. Specifically, 54.4% (n = 49) of participants actively used the QR code interaction, which enhanced their engagement with AI-generated reports and recommendations. The other 45.6% (n = 41) relied on core system functions without interactive features, indicating that while digital enhancements can improve the user experience for some clinicians, the essential AI-CDSS functions remain valuable even without interactive options. This suggests the system design effectively supports different levels of technological engagement among healthcare professionals (Figure 2f).

## 4. Discussion

The results of this cross-sectional survey provide compelling evidence for the practical application and acceptance of AI-driven CDSS in the management of bacteremia. Results demonstrated near-universal acceptance among physicians and high concordance with their own clinical judgment. The complete recognition (100%) of OneChoice’s clinical value by physicians surpasses the often mixed reception of other healthcare AI applications. Recent systematic reviews of AI-based decision-making systems in healthcare have revealed heterogeneous outcomes across various medical fields, with consistent benefits observed in areas such as depression treatment and pain management. Effects in other clinical sectors, however, remain diverse [13]. The finding that 97.8% of physicians found therapeutic decision-making easier is consistent with broader evidence on the use of machine learning in infectious diseases, where approximately 40% of systems are developed for intensive care settings and 25% for infectious disease consultations. These systems demonstrate tangible clinical benefits, including sepsis prediction algorithms that have reduced mortality rates by 30–60% in quasi-experimental studies and 58% in randomized controlled trials [8]. The high congruence rate (87.8%) between OneChoice’s recommendations and physicians’ therapeutic decisions indicates a strong correlation between AI-generated recommendation and physicians’ own clinical judgment. Notably, stratification by specialty revealed that non-infectious disease specialists demonstrated concordance rates of 89–100%, while infectious disease specialists showed lower concordance (33.3%), suggesting that the AI-CDSS may be particularly valuable for clinicians without specialized infectious disease training. This finding aligns with evidence from other clinical domains, where AI decision support tools have been shown to reduce disparities in diagnostic and therapeutic accuracy between general practitioners and specialists. In a previous study, we demonstrated the high correlation (80%) between recommendations that relied solely on molecular lab result data and recommendations that incorporated both phenotypic data and molecular data. Therefore, it is easy to postulate that this tool can be used throughout a patient’s clinical course.

The impressive 68.9% implementation rate of treatment changes based on OneChoice’s recommendations underscores a significant clinical impact that extends beyond simple acceptance, demonstrating that AI-driven guidance leads to tangible therapeutic adjustments. This rate is critical when considered in the context of the global challenges that antimicrobial stewardship programs face. In Latin America, data indicates that merely 46% of hospitals in Central and South America have adopted antimicrobial stewardship programs, with major obstacles including a shortage of dedicated pharmacists (63%), the lack of treatment guidelines tailored to local epidemiological data (33%), and insufficient microbiology lab capabilities (12%). The AI-driven CDSS assessed in our study directly addresses these resource challenges. It offers standardized, evidence-based recommendations. These recommendations can be customized to fit local epidemiological patterns and resistance profiles. This is particularly important given that 30–50% of the population in many Latin American nations depends on underfunded public healthcare systems [14]. The proven effectiveness of this system is especially pertinent when juxtaposed with findings that show infectious disease consultations, although linked to decreased mortality in Gram-negative bloodstream infections (adjusted hazard ratio 0.82, 95% CI 0.77–0.88), display significant variability across hospitals (2.7–76.1%) due to constraints in resources and specialist availability [13].

These findings hold significant clinical relevance, as highlighted by the current international guidelines from the Surviving Sepsis Campaign. These guidelines clearly acknowledge that machine learning outperforms traditional screening methods like SIRS (AUROC 0.70), MEWS (AUROC 0.50), and SOFA (AUROC 0.78) in identifying sepsis [15]. Moreover, the guidelines emphasize the urgent need for prompt antimicrobial treatment, with observational studies indicating that each hour of delay is associated with a 1.04-fold increase in the odds of in-hospital mortality [15]. The fusion of AI-driven clinical decision support systems with swift molecular diagnostics, as exemplified by the OneChoice platform, which offers therapeutic guidance 29 h sooner than traditional phenotypic methods [10], suggests a revolutionary leap forward in managing bacteremia. A 2017 systemic review found that rapid molecular diagnostic testing significantly reduces mortality risk and shortens time to effective therapy by about 5.03 h [6]. Conversely, delaying appropriate antibiotic treatment results in a roughly 20% increase in in-hospital mortality, a 70% increase in hospital length of stay, and a 65% increase in total inpatient costs, irrespective of antimicrobial resistance status [3].

Our research aligns with the growing body of evidence on AI technologies specifically designed to address antimicrobial resistance. Recent studies indicate that AI-powered diagnostic tools can process vast amounts of data with greater precision than humans, enabling quicker, more precise diagnoses through the use of Convolutional Neural Networks (CNNs) for microscopic image analysis and machine learning algorithms for genomic data analysis [16,17]. Current machine learning applications in antimicrobial resistance encounter significant hurdles, particularly the scarcity of high-quality, well-annotated, and standardized data, which is critical for training accurate and dependable AI models [16,17].

Furthermore, emerging evidence suggests that AI-powered clinical decision support systems can help bridge expertise gaps between specialists and generalists in infectious disease management. Studies have shown that AI systems demonstrate particular value for non-infectious disease specialists, with the most pronounced improvements observed in respiratory and internal medicine specialties [18]. Machine learning algorithms for antimicrobial selection have achieved coverage rates comparable to those of clinicians (85.9% vs. 84.3%), while enabling more targeted antibiotic therapy [19]. This standardization potential is particularly relevant given the significant variability in infectious disease consultation availability across hospitals (2.7–76.1%), suggesting that systems like OneChoice may help democratize specialized knowledge and support equitable antimicrobial stewardship across diverse healthcare environments [20].

Nevertheless, despite these promising results, certain limitations in AI performance must be acknowledged. Predictive algorithms for multidrug-resistant bacteremia still show only moderate discrimination (AUROC ≈ 0.70) and can generate clinically relevant false-negative results, a problem exacerbated in low-incidence or resource-limited settings where blood-culture contamination and low positivity rates add further uncertainty [21,22]. In our study, the 12.2% rate of discordant cases—mostly Pseudomonas aeruginosa and Enterococcus faecalis—illustrate how current models may underperform when key patient-level factors such as immunosuppression, renal impairment, or prior multidrug-resistant colonization are not incorporated [23]. A central challenge for future refinement is therefore to enhance the integration of contextual clinical information while preserving algorithmic generalizability across institutions, given that external validations frequently reveal performance degradation when models are applied to populations with distinct epidemiology or data infrastructures [24]. Indeed, independent evaluation of other clinical AI tools, such as the Epic Sepsis Model (AUROC 0.63 versus 0.76–0.83 initially reported), underscores this risk and highlights the need for continuous recalibration [25]. In addition, algorithmic bias and limited transparency persist as barriers to clinician trust and equitable care delivery, emphasizing the importance of explainability and robust bias-mitigation strategies [26]. Future iterations of AI-CDSS should therefore prioritize the inclusion of critical patient-specific variables, adopt multicenter validation frameworks such as SPIRIT-AI and CONSORT-AI to demonstrate external validity, and implement continuous-learning mechanisms capable of updating recommendations in response to evolving local resistance patterns [27,28].

When interpreting our findings, several limitations are worth noting. First, this cross-sectional survey design relies on physician self-reported perceptions and intended behaviors rather than directly observed clinical practices, introducing potential response bias and social desirability bias. Physicians may have overestimated their agreement with AI recommendations or their willingness to modify therapy based on CDSS guidance. Second, the study offers insights into physicians’ perceptions but does not directly assess outcomes, clinical effectiveness, or actual implementation rates in practice. Third, although the study population is diverse in terms of medical specialties, the research was conducted at a single institution in Lima, Peru, which may limit its generalizability to other healthcare environments with varying resources, patient demographics, or resistance patterns. Fourth, the potential for clustering effects due to multiple surveys completed by individual physicians may affect the precision of our estimates. However, we have addressed this through cluster-adjusted confidence intervals.

The 12.2% discordance rate between AI recommendations and physician choices highlights specific scenarios where the current AI model may benefit from further refinement. Specifically, cases involving *Enterococcus* species, complex polymicrobial infections, and immunocompromised hosts showed higher discordance rates, suggesting that the AI model would benefit from enhanced training data in these clinical contexts.

Furthermore, systematic reviews of AI healthcare applications have shown that only 42% of studies reported adverse events, and none reported an increase in adverse events due to AI interventions, underscoring the need for more thorough safety monitoring in future implementations [29]. The acceptance and clinical utility of AI-powered CDSS for managing bacteremia, as shown in this study, lay the groundwork for further research and implementation. Future research should focus on prospective clinical trials that evaluate patient outcomes, such as mortality rates, hospital stay durations, and antimicrobial stewardship metrics, to complement the encouraging data on physician acceptance presented here. Future investigations should focus on tackling the significant challenges in AI applications for antimicrobial resistance, particularly issues related to data quality and standardization that hinder the creation of accurate and reliable AI models [17].

## 5. Conclusions

This cross-sectional survey demonstrates exceptional clinical utility and physician acceptance of AI-powered CDSS in bacteremia management, with unanimous recognition (100%) of clinical value and substantial concordance (87.8%**;** Cohen’s κ = 0.76) between AI recommendations and physician therapeutic choices. Pathogen-stratified analysis revealed the highest concordance for *Escherichia coli* bacteremia (96.6%), the most common pathogen encountered. The meaningful implementation of treatment modifications in 68.9% of cases indicates that these systems effectively bridge critical gaps in infectious disease expertise and antimicrobial stewardship resources, particularly relevant given the 30% prevalence of extended-spectrum β-lactamase-producing organisms in our study population. These findings, combined with evidence that AI-powered systems can provide therapeutic guidance approximately 29 h earlier than conventional approaches while maintaining clinical appropriateness, position machine learning-based clinical decision support as a transformative tool for improving outcomes in bacteremia management.

For optimal clinical integration, we recommend that clinicians utilize AI-CDSS recommendations as an adjunct to clinical judgment rather than a replacement, particularly in complex cases involving immunocompromised hosts or polymicrobial infections. The AI-CDSS is especially valuable in settings with limited access to infectious disease consultation, for after-hours decision support, and as an educational tool for trainees and non-specialist physicians managing bacteremia. Future implementations should focus on developing mechanisms to provide real-time feedback and continuously improve recommendation accuracy. Areas requiring model enhancement include coverage of *Pseudomonas* and *Enterococcus* species, as well as the management of polymicrobial infections.

## Data Availability

The data analyzed in this manuscript, as well as its definitions, can be requested at any time.

## Supplementary Materials

The following supporting information can be downloaded at: https://www.mdpi.com/article/10.3390/life15111756/s1, Supplement S1: Onechoice Report; and Supplement S2: Onechoice Fusion report.
